# Extracellular RNA as a Versatile DAMP and Alarm Signal That Influences Leukocyte Recruitment in Inflammation and Infection

**DOI:** 10.3389/fcell.2020.619221

**Published:** 2020-12-18

**Authors:** Klaus T. Preissner, Silvia Fischer, Elisabeth Deindl

**Affiliations:** ^1^Department of Biochemistry, Medical School, Justus Liebig University Giessen, Giessen, Germany; ^2^Kerckhoff-Heart-Research-Institute, Department of Cardiology, Medical School, Justus Liebig University Giessen, Giessen, Germany; ^3^Walter-Brendel-Centre of Experimental Medicine, University Hospital, LMU Munich, Munich, Germany; ^4^Biomedical Center, Institute of Cardiovascular Physiology and Pathophysiology, LMU Munich, Munich, Germany

**Keywords:** extracellular nucleic acids, danger-associated molecular patterns, inflammatory vascular diseases, arteriogenesis, endothelial cells, virus infection, RNase1

## Abstract

Upon vascular injury, tissue damage, ischemia, or microbial infection, intracellular material such as nucleic acids and histones is liberated and comes into contact with the vessel wall and circulating blood cells. Such “Danger-associated molecular patterns” (DAMPs) may thus have an enduring influence on the inflammatory defense process that involves leukocyte recruitment and wound healing reactions. While different species of extracellular RNA (exRNA), including microRNAs and long non-coding RNAs, have been implicated to influence inflammatory processes at different levels, recent *in vitro* and *in vivo* work has demonstrated a major impact of ribosomal exRNA as a prominent DAMP on various steps of leukocyte recruitment within the innate immune response. This includes the induction of vascular hyper-permeability and vasogenic edema by exRNA via the activation of the “vascular endothelial growth factor” (VEGF) receptor-2 system, as well as the recruitment of leukocytes to the inflamed endothelium, the M1-type polarization of inflammatory macrophages, or the role of exRNA as a pro-thrombotic cofactor to promote thrombosis. Beyond sterile inflammation, exRNA also augments the docking of bacteria to host cells and the subsequent microbial invasion. Moreover, upon vessel occlusion and ischemia, the shear stress-induced release of exRNA initiates arteriogenesis (i.e., formation of natural vessel bypasses) in a multistep process that resembles leukocyte recruitment. Although exRNA can be counteracted for by natural circulating RNase1, under the conditions mentioned, only the administration of exogenous, thermostable, non-toxic RNase1 provides an effective and safe therapeutic regimen for treating the damaging activities of exRNA. It remains to be investigated whether exRNA may also influence viral infections (including COVID-19), e.g., by supporting the interaction of host cells with viral particles and their subsequent invasion. In fact, as a consequence of the viral infection cycle, massive amounts of exRNA are liberated, which can provoke further tissue damage and enhance virus dissemination. Whether the application of RNase1 in this scenario may help to limit the extent of viral infections like COVID-19 and impact on leukocyte recruitment and emigration steps in immune defense in order to limit the extent of associated cardiovascular diseases remains to be studied.

## Background

### DAMPs/Alarmins, PAMPs, and the Innate Immunity Response in Sterile Inflammation and Infection

Following cell stress, trauma, or exposure of the body to infectious or damaging factors, an immediate host response is mobilized by virtue of the innate immune system to recognize the external (pathogen-associated molecular patterns, PAMPs) or the body’s own (danger-associated molecular patterns, DAMPs) agonists and to provoke the release of alarm molecules, which is followed by a spatiotemporal and localized inflammatory response, including the release of cytokines (Tisoncik et al., 2012). The subsequent recruitment and accumulation of circulating leukocytes (neutrophils and monocytes/macrophages in a sequential order) to the site of inflammation culminates in their phagocytic action to remove cell debris and microbes with the help of the complement system. Both, intracellular killing (following phagocytosis of pathogens) and extracellular killing, which is carried out by neutrophils and involves “neutrophil extracellular traps” (NETs, de-condensed extracellular chromatin, a DNA-histone network on which neutrophil-derived components are concentrated), are vital parts of the innate host defense (Castanheira and Kubes, 2019). This is followed by the resolution of inflammation, including the recruitment of stem and endothelial cells, in order to restore tissue homeostasis. When DAMPs are cleared, the pro-inflammatory status of recruited leukocytes is changed to a reparative program, also directed by natural killer T cells (Van Kaer et al., 2013). As a consequence, neutrophils exit the site of inflammation by reverse transmigration back into the bloodstream (Zindel and Kubes, 2020). The concomitant tissue repair and regeneration process is achieved by platelet-dependent hemostasis and the blood coagulation machinery, resulting in temporary wound sealing by aggregated platelets and the formation of a stable fibrin network, which also prevents further entry of microorganisms (Kolaczkowska and Kubes, 2013).

**FIGURE 1:**
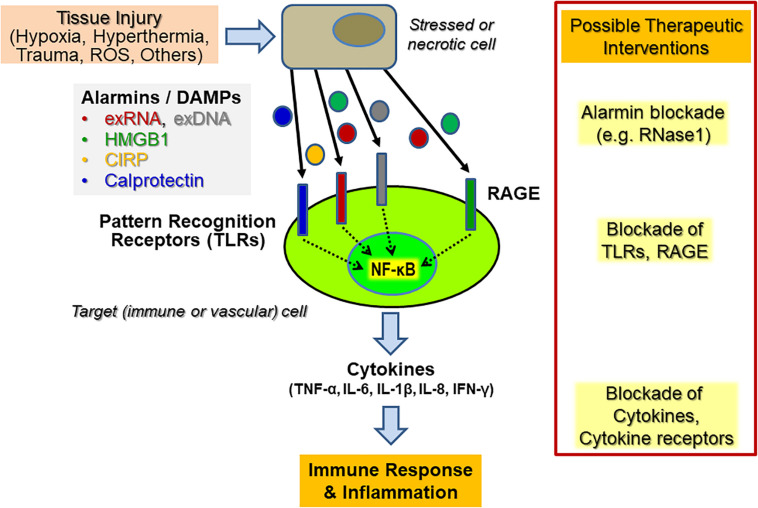
Endogenous cytokine release induced by alarmins upon sterile inflammation and potential targets for therapeutic interventions. Upon diverse types of cellular stress and activation of tissue and immune cells, a variety of alarmins or DAMPs (“Danger-associated molecular patterns”) such as HMGB1, extracellular RNA and DNA (exRNA, exDNA), CIRP (“Cold-inducible RNA-binding protein”) or calprotectin, are liberated and become recognized by various “Pattern recognition receptors” (PRRs), including Toll-like receptors (TLRs) and the “Receptor of advanced glycation end-products” (RAGE). Their subsequent signal transduction culminates in the promotion of, e.g., NF-κB activation in vascular cells and leukocytes (predominantly neutrophils and monocytes/macrophages) to trigger an enhanced cytokine release within the innate immune response, resulting in subsequent inflammatory reactions. Potential therapeutic targets for immunomodulation in both acute and chronic inflammatory diseases are alarmins, PRRs, and the induced downstream cytokines or cytokine receptors. Direct targeting can be achieved by inhibitory antibodies, by competitive inhibitors, by targeting the PRRs with antibodies or soluble decoy receptors. In the case of exRNA, RNase1 has been successfully used in preclinical animal models to prevent or inhibit the respective adverse outcome of inflammatory reactions.

Within the initial phase of innate immunity related to sterile inflammation, mediated by, e.g., hypoxia, hyperthermia, or oxygen radicals, the disturbed tissue homeostasis and cell damage is accompanied by the liberation of DAMPs or alarmins from necrotic cells or from activated immune cells (Figure 1). The structurally diverse and unrelated multifunctional alarmins include cytosolic, mitochondrial, or nuclear proteins (such as heat-shock proteins, histones, amphoterin/“high mobility group B1,” HMGB1 or neutrophilic calprotectin) as well as diverse self-nucleic acids (including nuclear DNA, ribosomal RNA, microRNAs) or heme and ATP. These DAMPs not only play an essential role inside cells prior to tissue injury but also serve multi-tasking functions (as “moonlighting factors”) outside cells by the activation of innate immune and vascular cells, including the recruitment of leukocytes and the sensing of antigen-presenting cells engaged in host defense and tissue repair (Bianchi, 2007). Upon uncontrolled release or overexpression, alarmins also play a pathophysiological role in a wide range of sterile or infection-induced immune and inflammatory disorders (Ehrchen et al., 2009; Andersson and Tracey, 2011). Alarmins may also induce the adaptive arm of the immune response via direct or indirect activation of antigen-presenting cells, including dendritic cells, thereby providing a relevant link between the innate and adaptive parts of the immune response (Bianchi and Manfredi, 2007). Hence, the diverse functional repertoire of alarmins renders them intriguing therapeutic targets, both, to reduce unwanted hyper-inflammation as well as to uncouple the innate and adaptive immune responses in chronic pathologies, including autoimmune disorders (Chan et al., 2012). Finally, alarmins may serve as useful diagnostic and prognostic biomarkers in inflammatory disorders.

### Pattern Recognition Receptors for PAMPs and DAMPs, Including Self-ExRNA

In situations arising from bacterial or viral infections, the host’s primary line of microbial recognition and pathogen sensing is made up of pattern recognition receptors such as cell membrane or endosomal Toll-like receptors (TLRs), many of which have also a key role in the detection of endogenous alarmins through signaling events associated with the induction of anti-inflammatory genes (Gong et al., 2020). More than 10 different TLRs exist, which are able to bind diverse exogenous infectious ligands classified as PAMPs (including bacterial DNA, lipopolysaccharide, flagellin, peptidoglycans, or viral double-stranded RNA). Other TLRs like TLR13 (only expressed in mice) recognize a conserved 10-nucleotide sequence from bacterial 23S ribosomal RNA that trigger immune responses, whereas TLR8 senses specific motifs in bacterial and mitochondrial RNAs (Kruger et al., 2015; Wang et al., 2016).

The body’s own alarmins, including nucleic acids originating from stressed or dying host cells, were found to induce pathological inflammatory responses by direct activation of specific TLRs to promote cellular signaling pathways: These involve either myeloid differentiation factor 88 (MyD88) or Toll- interleukin-1 receptor domain-containing adaptor-inducing interferon β (TRIF), both leading to the activation of transcription factors such as c-Jun N-terminal kinase or nuclear factor (NF)-κB. As a consequence, cytokines including tumor necrosis factor (TNF)-α, interleukin (IL)-1β or IL-6 will be released (Barrat et al., 2005; Yu et al., 2010; Leifer and Medvedev, 2016). The adaptor molecule MyD88 is involved in all signaling pathways activated by TLRs except for endosomal TLR3, which recognizes (viral) double-stranded RNA, single-stranded RNA, and also self-RNA fragments that mediate cellular activation through TRIF (Yamamoto et al., 2003). TLR10 was shown to play a role as an RNA-sensing receptor by binding to double-stranded RNA and regulating interferon-dependent responses (Lee et al., 2018). In another example, myocardial infarction was found to be attenuated in TLR3-deficient mice as a result of the activation of the TLR3-TRIF pathway by extracellular RNA (exRNA) released from damaged tissue, although the authors did not define the source and identity of the RNA (Chen et al., 2014). Alternatively, self-exRNA (mainly consisting of ribosomal RNAs) can induce pro-inflammatory activities to a large extent by TLR-independent mechanisms, which are poorly defined thus far (Preissner and Herwald, 2017) (see below). Finally, while microRNAs (miRNAs) can also serve as ligands of TLRs, the therapeutic administration of structurally similar short-interfering RNAs (siRNAs) may lead to undesirable activation of particular TLRs, such as the induction of inflammatory responses via TLR3 (Li and Shi, 2013; Pirher et al., 2017).

Moreover, the same TLR-dependent recognition machinery, either on the cell surface or on intracellular endosomes of host immune cells, appears to be responsible for recognizing endogenous alarmins together with exogenous factors in order to foster and maintain inflammatory reactions and to initiate their eventual resolution. However, it is not surprising that DAMPs and PAMPs may exhibit functional overlaps, influence each other, or even synergize in their functional activities, eventually provoking severe inflammatory disorders or chronic inflammation. In any event, the recognition of PAMPs by the host’s immune system is followed by the recruitment of leukocytes to the site of inflammation or infection with the release of diverse cytokines and the engagement of their phagocytic activities, culminating in the catching and killing of microbial invaders by neutrophilic granulocytes and macrophages. At this stage, NETs provide a functional scaffold in immune defense that drives (micro-)thrombosis as a principal mechanism of inflammation-hemostasis crosstalk (also designated “immuno-thrombosis”) that ultimately prevents the dissemination of microbes (Engelmann and Massberg, 2013). Following neutrophil apoptosis and the subsequent clearance of dying cells by macrophages (known as “efferocytosis”), the final resolution stage of these cells under physiological conditions is characterized by an anti-inflammatory cytokine signature (Kourtzelis et al., 2020).

### Scope

The aim of this review article is to provide an overview of the current state of the (patho-) physiological functions, particularly of ribosomal exRNAs as DAMP and alarmin, with special emphasis on their role in leukocyte recruitment as a central process in the innate immune response. Despite the fact that other extracellular RNA-species such as microRNAs and long non-coding RNAs do play an important role in inflammation and cardiovascular diseases as well (Weber et al., 2010; Heward and Lindsay, 2014), they would not be considered as DAMPs in the narrow sense and will not be dealt with here. Thus, ribosomal exRNAs with their multi-faceted roles as damaging factors in inflammation-driven diseases appear to be potential and challenging targets for therapeutic interventions using various approaches to antagonize exRNA-mediated pathophysiological actions (Sullenger and Nair, 2016; Bedenbender and Schmeck, 2020).

## The Multi-Step Process of Leukocyte Recruitment to the Site of Inflammation

**FIGURE 2:**
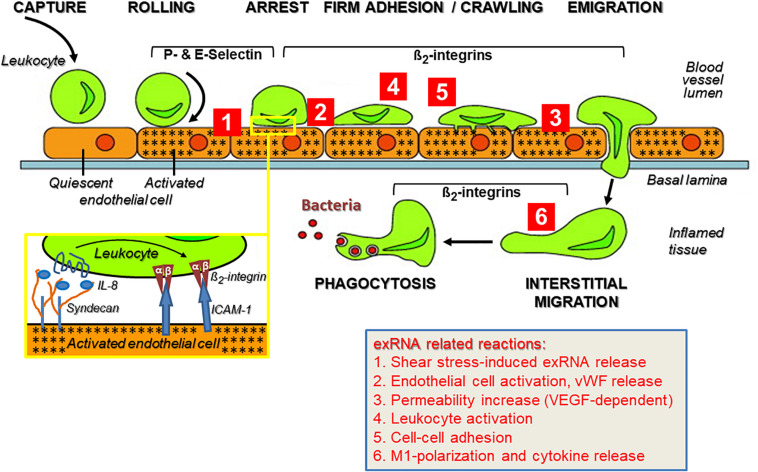
The multi-step recruitment of leukocytes to the source of inflammation and the influence of exRNA at multiple sites during this process. In an inflammatory situation, either initiated by microbial agonists or in their absence (sterile inflammation), leukocytes in the blood stream approach the inflamed vessel wall to find the shortest way toward the source of inflammation/infection in the tissue. Following their capture on the vessel wall, leukocytes start rolling on the inflamed endothelium (indicated by stars) due to blood stream-mediated mechanical forces together with weak adhesive interactions between leukocytes and endothelial P- and E-selectins. Based on the close approximation of both cell types, heparan sulfate proteoglycan (syndecan)-fixed chemokines (such as Interleukin-8) can reach and activate their leukocytic receptors (inset) as well as induce “inside-out” activation of β2-integrins with the result that the tethered leukocytes become firmly attached to the vessel wall via ICAM-1 and start crawling toward the nearest point of emigration. The transmigration phase along a chemotactic gradient is also supported by β2-integrins, as is the subsequent recognition of complement-activated bacteria by leukocytes for the ultimate phagocytosis reaction within the inflamed tissue. This route of consecutive steps of immune cell recruitment is taken by neutrophils (within hours) and monocytes/macrophages (within a few days) to execute a proper immune response. Modified from Schymeinsky et al. (2011). Ribosomal extracellular RNA (exRNA) is capable of influencing cellular interactions at multiple sites during leukocyte recruitment (indicated by red numbers): (Tisoncik et al., 2012; Castanheira and Kubes, 2019). Fluid shear stress in the circulation can mediate liberation of exRNA from endothelial cells, and exRNA induces vascular permeability in a VEGF-receptor-2/neuropilin-1-dependent fashion as well as the release of components stored in Weibel–Palade granules. (Van Kaer et al., 2013) exRNA induces activation of leukocytes to acquire an adhesive phenotype and to release cytokines that further promote immune cell activation (Kolaczkowska and Kubes, 2013; Zindel and Kubes, 2020). As a consequence, exRNA-primed leukocytes are further promoted to firmly attach to the inflamed endothelium as a prerequisite for their transmigration. (Bianchi, 2007) exRNA can induce M1-polarization of monocytes/macrophages and thereby provoke a significant pro-inflammatory cytokine release by these cells. The details on exRNA-cell interactions in this context are outlined in the text.

As part of the innate immune response, leukocyte recruitment is crucial in the pathways mediating sterile inflammation, infection, inflammatory disorders such as atherosclerosis, and autoimmune diseases like psoriasis, rheumatoid arthritis, vasculitis, or chronic lung diseases that involve a number of balanced cell-adhesive interactions between mobile blood cells and the activated endothelium (Muller, 2013; Kourtzelis et al., 2017). All of these pathophysiological situations are characterized by multiple forms of acute or chronic stress of cells and tissues, resulting in the appearance of a variety of DAMPs. A substantial increase in the levels of ribosomal exRNAs was observed in the circulation of patients (and preclinical animal models), and exRNAs were found concentrated at exposed sites in the vessel wall such as in atherosclerotic plaques (Simsekyilmaz et al., 2014; Zernecke and Preissner, 2016; Stieger et al., 2017). In any event, under the regimen of the innate immune system, leukocyte extravasation directs neutrophils within hours and macrophages within a few days to the extravascular site of inflammation or infection, comprising several cell-adhesive steps. A prerequisite for the leukocyte transmigration cascade, prior to the described cell–cell interactions, is the breakdown of the vessel wall permeability barrier by vasoactive mediators such as histamine that are generated via activation of perivascular mast cells and the complement system (Petri and Sanz, 2018). This allows the extravasation of blood solute and proteins near the site of inflammation before leukocytes initiate their rolling and extravasation process (Figure 2).

### Initial Selectin-Dependent Rolling of Leukocytes at the “Inflamed” Apical Side of the Vascular Endothelium

The motility of leukocytes in the bloodstream is slowed down near a locus of inflammation by their selectin-mediated rolling along the vessel wall in order to prepare these mobile cells for their transmigration toward the site of inflammation. Rolling interactions last seconds and are transient and reversible under conditions of blood flow due to the weak binding of P- and E-selectin to their common ligand, S-Lewis-x antigen. This is an oligosaccharide present in glycolipids and various glycoproteins on the surface of all mobile and stationary vascular cells. The expression of P- and E-selectins on the apical side of activated endothelial cells is a tightly regulated process that depends on the inflammatory conditions: Upon activation of the endothelium by various stimuli, including exRNA, P-selectin is immediately translocated from its storage site, the Weibel–Palade granules that serve as a vascular “emergency kit” (which also harbor IL-8, von Willebrand factor, tissue plasminogen activator, and RNase1), to the luminal endothelial cell surface (Fischer et al., 2011; Schillemans et al., 2019). This process allows the normally non-sticky and non-thrombogenic vessel wall surface to capture the patrolling leukocytes out of the bloodstream, providing a cell-to-cell approximation that is necessary for the subsequent leukocyte activation step mediated by chemokines. If the inflammatory stimulation of the endothelium continues for a longer period of time, E-selectin is expressed *de novo* on the apical side of the endothelium as well to augment leukocyte rolling.

### Chemokine-Induced Leukocyte Activation and Integrin-Mediated Firm Adhesion of Immune Cells

In addition to functioning as a kind of brake, rolling interactions along the vessel wall allow neutrophils to sense chemokines (such as IL-8, which is released during the initial exocytosis from endothelial Weibel–Palade granules) that are tightly associated with the apical endothelial glycocalyx by ionic interactions via heparan sulfate proteoglycans. Other chemo-attractants (including complement anaphylatoxin C5a, leukotriene LTB4, platelet activating factor, or bacteria-derived formylated peptides) derived from activated mast cells and tissue macrophages, and vasoactive agents such as histamine, released in the very early phase of the innate immune response, help to induce rapid neutrophil adhesion. This is achieved by converting the low-affinity, selectin-mediated interaction into a high-affinity, integrin-dependent firm arrest (Ley et al., 2007). The firm adherence of leukocytes to endothelial cells adjacent to the locus of inflammation is mediated by leukocyte integrins such as VLA-4 (α4β1), α4β7-integrin, Mac-1 (αMβ2), and LFA-1 (αLβ2) and their endothelial counter-receptors of the immunoglobulin superfamily, including intercellular adhesion molecules (ICAMs) as well as vascular cell adhesion molecule-1 (VCAM-1), all of which had been upregulated on the inflamed endothelium prior to leukocyte adherence (Yonekawa and Harlan, 2005). In this regard, exRNA serves as one of the endothelial cell-activating agonists to promote integrin- rather than selectin-dependent leukocyte adhesion, as observed in the cremaster vascular inflammation model (Fischer et al., 2012). While VCAM-1 interacts with leukocytic VLA-4, both ICAM-1 and ICAM-2 predominantly bind to β2-integrins (including LFA-1 and Mac-1) on leukocytes.

Chemokine-induced leukocyte adherence is primarily regulated via conformational changes and clustering of the indicated integrins through “inside-out” signaling, particularly involving several GTPase-dependent pathways (Rot and von Andrian, 2004; Wittchen et al., 2005). Integrin-mediated adhesion is relevant for neutrophil extravasation and the immune response as demonstrated by studies that utilized either mice that were deficient in one or more leukocyte integrin or patients with “Leukocyte Adhesion Deficiency” (LAD I) syndrome who lack functional β2-integrins (Springer et al., 1984; Ding et al., 1999; Yonekawa and Harlan, 2005). Neutrophil adhesion to the inflamed endothelium is reinforced by integrin-mediated “outside-in” signaling as a result of receptor clustering and conformational changes due to integrin ligation by multivalent adhesive protein ligands such as fibronectin or collagens. In addition to ICAMs on the activated endothelium, the DAMP binding protein “Receptor for advanced glycation end-products” (RAGE) has been identified as a receptor particularly for leukocytic Mac-1 under strong inflammatory conditions as in diabetes. Thus, in a preclinical model of inflammation, only the inhibition of both ICAM-1 and RAGE resulted in the total blockade of leukocyte arrest and transmigration (Chavakis et al., 2003). Finally, alternative integrin activation signals appear to be operative as well in the fine-tuning during the early arrest phase of neutrophils (but not monocytes), since both zinc ions as well as the glycolipid-anchored urokinase receptor were shown to provide an essential contribution to integrin activation, as demonstrated in preclinical animal models (May et al., 1998; Chavakis et al., 1999).

### Trans-Endothelial Migration of Leukocytes

After their firm adhesion, leukocytes crawl over the endothelial cell surface, involving their integrins Mac-1 and LFA-1 until they reach the nearest junction appropriate for transmigration (Schenkel et al., 2004). Trans-endothelial migration (also designated “diapedesis”) primarily takes place at the intercellular junctions in a para-cellular manner, whereby tricellular junctions as well as endothelial junctions positioned above thin-layer basement membranes have been proposed as preferential sites for transmigrating neutrophils *in vivo* (Burns et al., 1997; Wang et al., 2006). Alternatively, a minority of neutrophils and other leukocytes (about 5–10%) may enter the extravascular tissue via a transcellular route, i.e., through the endothelial cells, particularly when intravascular locomotion is disabled *in vivo* (Shaw et al., 2004; Nourshargh and Marelli-Berg, 2005; Phillipson et al., 2006). In essence, the preferred routing of emigrating leukocytes may depend on the level of the pro-inflammatory stimuli as well as the type of blood vessel (wall). In addition to their importance in leukocyte-endothelial adherence, ICAM-1 and ICAM-2 also participate in leukocyte trans-endothelial migration (Shang and Issekutz, 1998).

The subsequent sub-endothelial interstitial migration of leukocytes into the inflamed tissue is predominantly mediated by β1-integrin family members as well as by the β2-integrin Mac-1 that also binds to fibrin(ogen) in the wound matrix. This process is facilitated by leukocyte-associated or secreted proteases as well as by glycosaminoglycan-degrading enzymes, which help the neutrophils and macrophages to invade the extracellular matrix (Stamenkovic, 2003). After leukocytes have terminated their emigration, leaks in the vessel wall are prevented by contractile actin filaments surrounding the diapedesis pore, keeping this opening tightly closed around the transmigrating neutrophils, and platelets interacting with endothelial von Willebrand factor activate endothelial Tie-2 receptors to secrete angiopoietin-1, thereby preventing diapedesis-induced leakiness (Duong and Vestweber, 2020). Finally, leukocytes migrate along the sub-endothelial side, traverse the basement membrane, and invade the inflamed tissue toward the gradient of chemotactic activity (e.g., activated mast cells, complement-related anaphylatoxins), where they start their program of defensive actions as phagocytic cells to remove invading microbes and cell debris (Underhill et al., 2016; Voisin and Nourshargh, 2019). Although exRNAs bind to heparin-binding growth factors such as “Vascular endothelial growth factor” VEGF (Fischer et al., 2007), it remains to be demonstrated whether the extracellular matrix-bound ribonucleic acids could serve as a guidance cue for migrating phagocytes toward their destiny.

### Contribution of Cell Adhesion Receptors to Leukocyte Diapedesis

Along the para-cellular diapedesis pathway, endothelial junctions are the major barriers for transmigrating leukocytes, whereby several types of junctions are involved (Bazzoni and Dejana, 2001): (i) Adherence junctions (*zonula adherens*) that mediate cell–cell contacts via homophilic, calcium-dependent binding between adjacent VE-cadherin molecules; (ii) the most apical tight junctions (*zonula occludens*), which form a close intercellular adhesive web that consists of three types of transmembrane proteins; and (iii) junctional adhesion molecules (JAMs), which are linked intracellularly to cytoskeletal signaling proteins such as zonula occludens-1 and which can form cell–cell contacts by homophilic interactions and can act as receptors for leukocyte integrins (Weber et al., 2007). As demonstrated *in vitro* by cell culture experiments as well as *in vivo* (brain edema), exRNA can disturb the integrity of the vessel wall by disconnecting cell–cell junctions in an irreversible fashion, as compared to thrombin which acts in a temporary manner as a permeability-increasing factor (Fischer et al., 2007, 2014).

A well-known adhesive component in leukocyte transmigration is platelet endothelial cell adhesion molecule-1 (PECAM-1), which is expressed both on platelets and leukocytes as well as at the inter-endothelial cell junctions. Like JAMs, it can interact in a homophilic as well as a heterophilic fashion to regulate leukocyte trans-endothelial migration (Sullivan and Muller, 2014). Here, PECAM-1 may recycle in vesicular structures between the junctions and the sub-junctional plasma-lemma and is thereby targeted to the sites of the vessel wall where leukocyte transmigration takes place. Altogether, the variable interactions of these transmembrane adhesion molecules, whose expression pattern differs at the leading and the trailing edges of each emigrating leukocyte as well as within the endothelial cell clefts, guide the transmigrating cells within minutes from the luminal to the basolateral side; however, their precise molecular coordination still remains a mystery (Vestweber, 2015; Duong and Vestweber, 2020).

## exRNAs as Ubiquitous Damps and Pro-Inflammatory Factors

More than 50 years ago, extracellular nucleic acids were identified in blood plasma and in extracellular body fluids as well as in cell supernatants, and their appearance was found to be associated with some disease states (Mandel and Metais, 1948). For example, RNA-proteolipid complexes and also free exRNA were initially identified in cancer patients and were proposed to mediate host-tumor interactions (Wieczorek et al., 1985; Kopreski et al., 1999, 2001). Basic research as well as clinical studies have provided experimental evidences that the body’s own extracellular nucleic acids are important players in the crosstalk between immunity and cardiovascular pathologies or other diseases. Following stress- or injury-induced liberation, these endogenous polyanionic macromolecules not only serve as alarmins/DAMPs or biomarkers of, e.g., cell necrosis, rather, their functional repertoire reaches far beyond their activities in innate immunity. In fact, (patho-) physiological functions of exRNAs and of extracellular DNA (exDNA) as well are associated with and in many cases causally related to, e.g., arterial and venous thrombosis, atherosclerosis, ischemia/reperfusion injury, or tumor progression, especially in association with the elevated inflammatory status of these diseases (Fischer et al., 2007, 2012, 2013; Kannemeier et al., 2007; Simsekyilmaz et al., 2014; Cabrera-Fuentes et al., 2015b). However, many of the underlying molecular mechanisms are far from being completely understood. Interestingly enough, novel *in vitro* and *in vivo* approaches, including natural endonucleases or synthetic nucleic acid binding/neutralizing polymers as antagonists, seem to be promising and safe therapeutic options for future investigations to combat the damaging nature of exRNA or exDNA (Preissner and Herwald, 2017; Naqvi et al., 2018; Thålin et al., 2019).

In the context of innate immunity, both in sterile inflammation as well as with regard to infectious conditions, exRNAs constitute typical DAMPs, which are released during tissue damage by either active or passive processes (Vénéreau et al., 2015) or following bacterial and viral infections (Chen et al., 2017; Kawasaki and Kawai, 2019). Depending on their size, composition, and complexity, exRNAs are involved in the typical recognition by membrane-bound PRRs as outlined above, including TLRs or RAGE (Bertheloot et al., 2016), as well as cytosolic receptors including retinoic acid-inducible gene I, melanoma differentiation-associated protein 5, or cyclic GMP-AMP synthase (Roers et al., 2016). Binding of exRNAs by such PRRs leads to the induction of different signaling pathways that result in the activation of transcription factors like c-Jun-N-terminal kinase or NF-κB and the subsequent release of cytokines including TNF-α, IL-1β, or IL-6 (Yu et al., 2010; Leifer and Medvedev, 2016). Ribosomal-type exRNAs (which constitute the majority of exRNAs in plasma) (Cabrera-Fuentes et al., 2015b) are different from other nucleic acid DAMPs, in that they fulfill a number of additional extracellular functions independent of recognition by PRRs, particularly related to the onset and progression of different cardiovascular diseases as will be outlined below.

### Types of ExRNAs and Their Characteristics

Extracellular RNAs are a heterogenous group of ribonucleic acids, including small RNAs (e.g., miRNAs), mRNAs, tRNAs, ribosomal RNAs, and long non-coding as well as circular RNAs, each of which has different (extracellular) functions and a different impact on the respective cells and tissues in their microenvironment, either alone or together with other molecules. ExRNAs can be liberated from cells in a free form or bound to proteins or phospholipids as well as in association with extracellular vesicles (EVs) or apoptotic bodies (Valadi et al., 2007; Zernecke et al., 2009; Arroyo et al., 2011; Vickers et al., 2011; Creemers et al., 2012). Some mechanisms of exRNA biogenesis and its vesicular loading have been described elsewhere (Patton et al., 2015; Abels and Breakefield, 2016; Pérez-Boza et al., 2018). Analyses of EV-associated exRNAs indicated that miRNAs together with ribosomal RNAs form the majority of this fraction; yet, on a weight basis, ribosomal RNAs are the far most abundant type of exRNA in human blood plasma (Crescitelli et al., 2013; Danielson et al., 2017). The association of exRNA with various proteins or with ribonucleoprotein complexes as well as the binding to high-density lipoproteins not only provides protection of exRNAs from degradation by extracellular RNases, but these interactions may modulate the reactivity and immunogenicity of the respective binding partners (Wieczorek et al., 1985; Fischer and Preissner, 2013; Jaax et al., 2013). While only low levels of circulating exRNA can be detected in extracellular fluids under quiescent conditions *in vivo* and *in vitro* (<100 ng/ml), under conditions of cell activation or tissue injury such as hypoxia, infection, inflammation, or tumor growth the concentration of self-exRNAs can increase dramatically (Rykova et al., 2012; Zhou et al., 2019).

The majority of self-exRNAs (including microRNAs and ribosomal RNAs) is released in association with EVs by a wide range of cells under shear stress or following different types of stimulation by endogenous or exogenous inflammatory and other agonists (Lasch et al., 2019; O’Brien et al., 2020). EVs are divided into three subclasses depending on their site of origin inside the cells they are derived from: apoptotic bodies (large EVs: 800–5000 nm) are released from cells undergoing apoptosis; micro-vesicles (medium-sized EVs: 100–1000 nm) are released by budding from the plasma membrane; exosomes (small EVs: 30–150 nm) originate from the internal surface of multi-vesicular bodies in the endosomal compartment of cells (Kim et al., 2017). In particular, these nano-sized bodies provide intercellular communication functions by delivering intracellular material (such as miRNAs and mRNAs) to target cells and fulfilling regulatory functions by altering cell activities (Dinger et al., 2008; Ekström et al., 2012). These aspects are of particular relevance for exosome-delivered miRNAs, whose main intracellular functions are to regulate gene expression and translational processes in target cells, including those related to pathological processes in cancer, cardiovascular diseases, or autoimmune disorders (Kim et al., 2017). In contrast, ribosomal exRNAs, which constitute the majority of exRNA, are believed to fulfill a variety of functions outside of cells that will be discussed in more detail.

Upon microbial infection, with the appearance of viral or bacterial nucleic acids, several mechanisms exist to prevent a (counterproductive) activation of PRRs by self-nucleic acids. Generally, endosomal TLRs respond to double- or single-stranded viral or bacterial RNAs and DNAs, whereas cell-surface TLRs recognize a variety of other accessible microbial patterns. Viruses and bacteria typically enter the cells via endocytosis or phagocytosis, whereby their DNAs or RNAs are protected from degradation, e.g., by capsid proteins, until they are released within the endo-lysosomal compartment of host cells to trigger TLR-dependent signaling pathways. In contrast, self-extracellular nucleic acids are degraded by extracellular nucleases unless they are protected in association with nucleoprotein complexes or cell membranes (Marek and Kagan, 2011). Thus, under physiological conditions, self-nucleic acid-related signaling may be limited by nuclease activities, thus preventing their detection by nucleic acid sensors.

### Analysis of Self-ExRNAs

Since exRNA-containing EVs are found in most body fluids, their potential use as biomarkers for particular disease conditions has been explored, and the “National Institutes of Health” and other institutions^1^ are evaluating the use of exRNAs, in particular miRNAs, as biomarkers for various human pathologies (Quinn et al., 2015; Das et al., 2019). The easy access and stability of EV-miRNAs in biological fluids makes them potentially suitable for use as diagnostic biomarkers, and in patients, different miRNA profiles implicate their participation in disease pathogenesis (Bellingham et al., 2012; Gui et al., 2015; Lugli et al., 2015). For example, the nature of a particular exRNA species contained in EVs can be assessed by RTqPCR (Bellingham et al., 2012; Gui et al., 2015; Lugli et al., 2015). The composition of exRNA is further analyzed by capillary electrophoresis and RNA sequencing (Tanriverdi et al., 2016; Yuan et al., 2016; Saugstad et al., 2017). Other methods, either qualitative/semi-quantitative or quantitative, depending on the respective reagent, utilize the specific reaction of fluorescent dyes with exRNA (Jones et al., 1998; Wu et al., 2020). Furthermore, methods currently available to isolate exRNA in biofluids have been compared and described previously, including the characteristics of exRNA-loaded EVs. They can be monitored by intra-vital microscopy and immunohistochemistry, provided EVs are fluorescently labeled via expression of palmitoylated green-fluorescent protein in donor cells (van der Vos et al., 2016; Small et al., 2018). Yet, the heterogeneity of exRNAs together with their presence in different cellular fractions require further modes of analyses and interpretations. In particular, the association of exRNA with multiple subtypes of EVs as well as with ribonucleoproteins and lipoprotein complexes or the formation of soluble vesicle-free supramolecular complexes with proteins, which are RNase-resistant, may complicate the analysis of exRNA (Tosar and Cayota, 2018; Das et al., 2019).

## exRNA and Leukocyte Recruitment in the Context of Innate Immunity

Although several subtypes of exRNA, including miRNA, long non-coding RNA, and ribosomal exRNA, have been described in the context of inflammatory cell signaling, the following paragraphs will focus on exRNAs, mostly consisting of ribosomal RNA, as a direct/indirect DAMP in inflammatory situations that influences the different steps of leukocyte recruitment. This includes the role of exRNA as (i) an ubiquitous alarmin, (ii) a cofactor for bacteria-host cell interaction and microbial invasion, (iii) a hyper-permeability factor that induces vasogenic edema, (iv) a promoter of leukocyte interaction with the inflamed endothelium, (v) an inducer of cytokine release from macrophages, (vi) an important mediator of arteriogenesis, and (vii) a potent pro-thrombotic cofactor.

### ExRNA as an Alarmin and DAMP and Recognition of Self- and Non-self-ExRNAs

**FIGURE 3:**
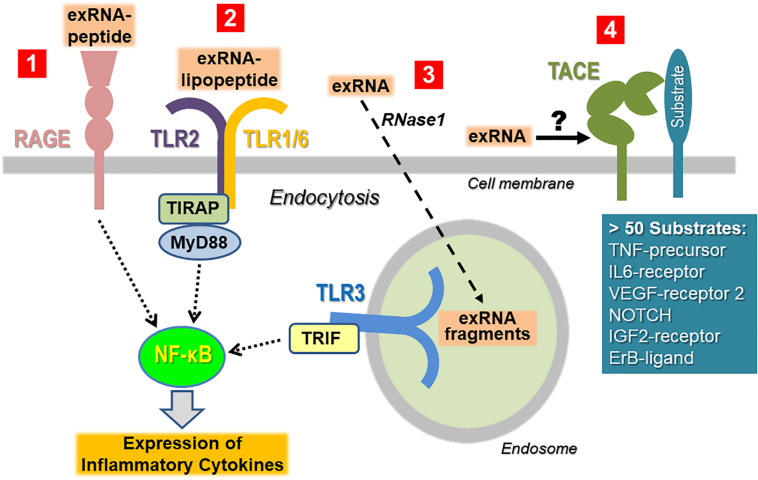
Cellular recognition of exRNA by various unrelated cell surface proteins/receptors. Once the concentration of exRNA has reached a certain level upon cell necrosis or other stress situations, the ribonucleic acid can induce activation of immune and tissue cells either in isolated, in complexed form as indicated, or in association with extracellular vesicles. To simplify the illustration, the latter case has not been depicted (Tisoncik et al., 2012). ExRNA-polypeptide complexes are recognized by RAGE, which also serves as receptor site for other DAMPs and PAMPs (Castanheira and Kubes, 2019). Likewise, in complex with certain PAMPs such as lipopeptides, exRNA can be recognized in a synergistic way in a TLR2-dependent fashion and thereby augments the immune cell activation of the PAMP (Van Kaer et al., 2013). Macromolecular exRNA, which can be degraded by extracellular RNase1, can be taken up by cells and promotes cellular activation via endosomal TLR3 under certain conditions. All of these pathways culminate in activation of NF-κB-dependent signaling, ultimately leading to the expression and liberation of cytokines, often in excess to provoke hyper-inflammation (Zindel and Kubes, 2020). ExRNA can potentially trigger the activation of the sheddase “TNF-α converting enzyme” (TACE) to induce the proteolytic cleavage of cell surface-bound protein substrates such as the precursor form of TNF-α in order to release the active cytokine. Since TACE can act as a sheddase for a number of cell-bound substrates, it is proposed that exRNA can trigger this protease to generate a variety of (inflammatory) products at multiple sites in the body.

To minimize the recognition of self-nucleic acids, the binding of non-self nucleic acid ligands to the signaling receptors (particularly belonging to the TLR family) will induce their sequestration away from the cell surface in the cytoplasm or in endosomes. Thus, isolated ribosomal exRNA appears not to be recognized by TLRs expressed on the cell surface in a classical way, unless prior modification or complexation of the ligand takes place. When self-nucleic acids are highly abundant, e.g., as a result of cell damage, defects in the degradation or processing machinery of nucleic acids, or in the binding and cellular uptake into endosomes, self-nucleic acids may activate endosomal PRRs (Bertheloot et al., 2016; Roers et al., 2016; Miyake et al., 2017). Self-exRNA may also reach endo-lysosomes and PRRs if bound to proteins or manipulated by transfection agents (Figure 3).

Autoimmune diseases like psoriasis serve as an example of how self-exRNA may play a role in immune responses. Complexes of self-exRNA and the antimicrobial peptide LL37 were shown to be recognized mainly by TLR7 and TLR8 to promote an autoimmune response (Zakrzewicz et al., 2016). LL37 constitutes the C-terminal portion of human cathelicidin from which LL37 is proteolytically released during immune cell activation. It serves as basic immune-modulatory peptide, and by forming complexes with polyanionic exRNA and exDNA it prevents their degradation. Together with exRNA derived from necrotic cells, LL37 activates “mitochondrial antiviral-signaling protein” and induces production of, e.g., interferon (IFN)-β to provoke maturation of dendritic cells (Zhang et al., 2016). Moreover, the exRNA-LL37 complex is capable of activating TLR7-expressing dendritic cells, thereby triggering the secretion of IFN-α, but not IL-6 or TNF-α. Also, exRNA-LL37 complex-mediated activation of TLR8 can lead to differentiation of myeloid dendritic cells and the release of IL-6 and TNF-α. Thus, the cationic antimicrobial peptide LL37 may convert self-exRNA into a trigger of TLR7/TLR8 in human dendritic cells in psoriatic lesions, allowing the initiation and progression of this autoimmune response (Ganguly et al., 2009). Interestingly enough, this complex was also found to trigger the TLR8/TLR13-mediated release of cytokines and NETs together with exRNA in neutrophils, thereby establishing a self-propagating vicious cycle that contributes to chronic inflammation in psoriasis (Herster et al., 2020).

There are other examples of the participation of self-and non-self-exRNA in immune responses. TLR7 (or TLR3), the classical receptors for small non-self-viral RNAs, appear not to be activated by exRNA alone in macrophage cell cultures, whereas a synergistic effect of exRNA together with the lipopeptide Pam2CSK4 on TLR2 activation was found, resulting in the synergistically elevated expression of cytokines in macrophages (Noll et al., 2017). Thus, certain isolated PAMPs are unable to mount a robust inflammatory response on their own unless self-exRNA (derived from trauma or necrosis) acts as a potent adjuvant. Moreover, in myocardial ischemia, cell-free exRNA has been proposed to promote apoptosis of cardiac muscle cells by activation of TLR3-TRIF signaling pathways (Chen et al., 2014). In this study, however, the nature of self-exRNA was not further defined, making it difficult to relate these results to our knowledge about self-exRNA-mediated pathways and their role in disease.

For endosomal TLRs in immune cells to be available for binding to non-self exRNAs upon infection and immune signaling, the access of self-nucleic acids from the extracellular environment to these TLRs needs to be limited. Here, cell-membrane expressed RAGE as a primary recognition site for glycated proteins fulfills functions as DAMP-receptor, for e.g., HMGB1, but has also been identified to promote the uptake of both exDNA and exRNA into endosomes. This may lower the immune recognition threshold for the activation of TLR9, the principal DNA-recognizing signaling receptor. Moreover, RAGE is important for the detection of nucleic acids *in vivo*, since mice deficient in RAGE were unable to mount an inflammatory response to exDNA in the lung (Sirois et al., 2013). In addition, RAGE was shown to bind self-exRNA in a sequence-independent manner to enhance cellular uptake of exRNA into endosomes. Gain- and loss-of-function studies demonstrated that RAGE increased the sensitivity of all single-stranded RNA-sensing TLRs (TLR7, TLR8, TLR13), indicating that RAGE appears to be an integral part of the endosomal nucleic acid-sensing system (Sirois et al., 2013).

### ExRNA Interactions With Microbes During the Process of Infection

The adherence to and invasion of eukaryotic cells and tissues by pathogenic bacteria are the main mechanisms for microbial colonization, evasion of immune defenses, survival and propagation, and the cause for infectious diseases in their mammalian hosts. Several structurally and functionally distinct “adhesins” of bacteria facilitate their specific recognition and their interactions with host cell surfaces (e.g., by integrins) and the extracellular matrix (e.g., by fibronectin or vitronectin) (Hammerschmidt et al., 2019). In tissue fluids, the binding of microbial components to (adhesive) host proteins may alter their structure or can influence cellular mechanisms that determine cell and tissue invasion of pathogens (Hammerschmidt, 2009; Hammerschmidt et al., 2019). In addition to host plasma proteins such as plasminogen or vitronectin which serve as essential virulence factors for the invasion of several Gram-positive bacteria, self-exRNA in a dose-dependent manner was found to increase the binding and uptake of *Streptococcus pneumoniae* in alveolar epithelial cells (Bergmann et al., 2009; Zakrzewicz et al., 2016). Extracellular enolase, a plasminogen receptor, was identified as an exRNA-binding protein on the *S. pneumoniae* surface, with several basic amino acid residues serving as exRNA-binding sites. Moreover, additional exRNA-binding proteins were identified in the pneumococcal cell wall using mass spectrometry. Due to the high number of such RNA-interacting proteins on pneumococci, treatment with RNase1 successfully inhibited exRNA-mediated pneumococcal alveolar epithelial cell infection (Zakrzewicz et al., 2016). These data support further efforts to employ RNase1 as an antimicrobial agent to combat pneumococcal infectious diseases.

### ExRNA and Vascular Permeability

The endothelium constitutes the inner lining of all blood and lymphatic vessels and functions as a natural barrier between the flowing blood or lymph and the underlying tissues. The balance in the exchange of molecules and mobile leukocytes across the vascular endothelium is maintained by several systemic as well as site-specific homeostatic transport mechanisms involving the dynamic contribution of junctional proteins and adhesion receptors, as indicated above. In response to a variety of (patho-)physiological stimuli, including histamine, thrombin, VEGF or activated neutrophils, endothelial cells immediately start to reorganize intercellular junctions to facilitate trans-endothelial flux resulting in an increased para-cellular leakage (Kumar et al., 2009). Likewise, edemagenic agonists as well as lipid mediators induce intracellular signaling pathways, involving different protein kinases as well as the cytoskletal machinery to disrupt cell–cell adhesion, resulting in hyper-permeability (Sukriti et al., 2014; Karki and Birukov, 2018). In fact, this scenario is a significant problem in vascular inflammation observed in a variety of pathologies, including, ischemia-reperfusion injury, sepsis, acute respiratory distress syndrome (ARDS) and others.

While the regulation and fine-tuning of endothelial barrier functions also involve the contribution of intracellular microRNAs (Cichon et al., 2014), platelet-derived EV-associated non-coding exRNAs (predominantly microRNAs) can functionally influence the integrity and other features of the endothelium, thereby providing a molecular communication between blood cellular components and the vessel wall (Randriamboavonjy and Fleming, 2018). In this regard, various exRNA species were shown to directly or indirectly disturb the permeability barrier of the endothelium by changing the expression profile of vasoactive molecules or altering the quality of cell-to-cell junctions (Fischer et al., 2007, 2014). Following the initial phase of DAMP- or PAMP-sensing mechanisms by host pattern recognition receptors (such as TLRs or RAGE) on immune and tissue cells, an immediate consequence is the release of a variety of cytokines, chemokines, and other factors (including exRNA) from mast cells, tissue macrophages, and endothelial cells. Thus, blood vessels in close proximity to the site of inflammation or the inflammatory agonists subsequently adopt an appreciable level of cellular activation to participate in the immune response. Here, endothelial cells are particularly prone to various stimuli and switch their phenotype from non-adhesive, non-thrombogenic to inflammatory, reflected by elevated cellular permeability, the expression of adhesion receptors (such as selectins, ICAMs), and the coating of their apical glycocalyx surface by chemokines to indicate this pro-inflammatory status. Together, these aspects are crucial in the primary phase of leukocyte attraction to the inflamed vessel wall, as previously outlined (Pober and Sessa, 2007; Petri et al., 2008).

In fact, ribosomal exRNA elicits an immediate and largely irreversible permeability-increasing activity in vascular endothelial cells *in vitro* that is associated with a robust rise in intracellular calcium ions; *in vivo* this is reflected by edema formation due to *Sinus sagittalis* thrombosis or stroke in animal models (Fischer et al., 2007; Walberer et al., 2009; Bálint et al., 2013). In particular, the exRNA-provoked increase in vessel permeability correlates with the disorganization of the tight and adherence junctional proteins zonula occludens-1, occludin, and VE-cadherin. Mechanistically, the permeability-enhancing function of exRNA is mediated by its direct high-affinity interaction with extracellularly bound VEGF, resulting in the activation of the VEGF-receptor-2/neuropilin-1 signaling complex that is reminiscent of the function of cell-surface heparan sulfate proteoglycans which act as co-receptors for VEGF (Fischer et al., 2009, 2014). This is followed by activation of phospholipase C and intracellular release of calcium ions, which together promote hyper-permeability of the endothelium. Also, fluid shear stress in blood vessels may lead to the release of endothelial cell-derived exRNA, which subsequently interacts (like heparin) with VEGF to promote vascular hyper-permeability in a VEGF-receptor-2/neuropilin-1-dependent fashion. In addition, locally enhanced VEGF-dependent signaling results in the exocytosis of Weibel–Palade granules (Lasch et al., 2019) to provide additional alarming molecules such as the chemokine IL-8, as will be detailed below for the process of arteriogenesis.

Due to its ability to induce cytokine release in monocytes/macrophages, exRNA also indirectly contributes to cytokine-mediated destabilization of the endothelium. Although direct activation of endothelial TLR3 (an endosomal receptor for non-self, double-stranded RNA) by ribosomal self-exRNA does not play a dominant role in vascular permeability changes (Fischer et al., 2009), synthetic virus-mimicking double-stranded RNA or the TLR3-agonists poly (I:C) caused a permeability increase of the blood-brain-barrier or provoked lung endothelial barrier dysfunction, respectively (Huang et al., 2016; Noda et al., 2018). It remains to be clarified, however, whether exRNA released *in situ* may also promote the activity of other vasodilators such as bradykinin, since exRNA was found to be a potent auto-activating cofactor for the precursor protein of this peptide, high-molecular-weight kininogen (HMWK), as well as of other proteins of the contact phase system of blood coagulation, including factors XI and XII (Kannemeier et al., 2007).

### ExRNA, Leukocyte Transmigration, and Inflammation-Based Pathologies

Besides the established inflammatory agonists mentioned above, the exposure of quiescent endothelial cells (*in vitro* as well as *in vivo*) toward ribosomal exRNA not only induces vascular permeability to allow the enhanced trans-endothelial trafficking of molecules, but also triggers the recruitment of inflammatory cells to the vessel wall. This was demonstrated *in vivo* using a murine cremaster muscle vasculature model (Fischer et al., 2012). The observed exRNA-induced expression of ICAM-1 on the endothelium, which was comparable in its extent to the agonistic action of TNF-α, reduced the selectin-dependent rolling of leukocytes and promoted their firm adhesion and extravasation by utilizing activated β2-integrins. Moreover, exRNA-induced leukocyte adhesion and transmigration was shown to depend on the activation of the VEGF-receptor 2 system and to be reinforced by exRNA-mediated release of cytokines such as monocytic TNF-α, which by itself already aggravates the inflammatory process. Furthermore, exRNA facilitated the acute hypoxia-induced leukocyte adhesion and infiltration in murine lungs through TLR-interferon-γ-STAT1 signaling pathways (Biswas et al., 2015). Since RNase1 was shown to significantly prevent the exRNA-provoked inflammatory outcome in the indicated preclinical animal models, the endonuclease might constitute a new type of therapeutic intervention for patients with inflammation-based diseases (see below).

The indicated functional relationships between exRNA and inflammatory responses has been corroborated in patients (presented with elevated plasma levels of exRNA) and especially in authentic animal models of atherosclerosis and rheumatoid arthritis, two established pathological scenarios of chronic inflammation. Under disease conditions, significantly increased levels of exRNA were found to be deposited at the typical sites of injury in atherosclerotic lesions as well as in affected joints in patients with rheumatoid arthritis (Simsekyilmaz et al., 2014; Zimmermann-Geller et al., 2016). Moreover, under conditions of ischemia-reperfusion injury, following intervention in occluded carotid arteries *in vivo*, exRNA was found to be a potent damaging factor, leading to cytokine (particularly TNF-α) release and cardiomyocyte death as well as to the accumulation of macrophages (Cabrera-Fuentes et al., 2014). The fatal situations in all of the *in vivo* models cited were prevented or dampened by degrading exRNA with the help of RNase1 administration, which thereby acts as an efficient anti-inflammatory and tissue-protective factor for improving the overall outcome (see below).

### ExRNA-Induced Release Reactions in Immune Cells (Macrophages, Mast Cells)

The levels of both exRNA and TNF-α were found to be concomitantly increased under the inflammatory conditions in the animal models discussed above as well as in human blood plasma in the transient perioperative ischemic situation associated with cardiac surgery (Cabrera-Fuentes et al., 2015b). The same elevation of these parameters was found in an ischemia-reperfusion injury model in mice and in isolated rat hearts, whereby cardiomyocytes could be identified as a major source of ribosomal exRNA. Functionally, exRNA and TNF-α appear to act in a feed-forward loop to promote cardiac ischemia-reperfusion injury: the increase in exRNA leads to an accumulation of TNF-α, and in turn, TNF-α activation of adjacent cells via TNF-receptor-1 provokes an increase in exRNA as well, resulting in a hyper-inflammatory situation (Cabrera-Fuentes et al., 2014). As an example, exRNA and TNF-α together induce the expression of reactive oxygen species (ROS) and inducible NO synthase or monocyte chemo-attracting protein (MCP)-1 to amplify the extent of inflammation (Cabrera-Fuentes et al., 2014).

*In vitro* studies using different tissue and immune cell cultures revealed the prominent exRNA-induced expression (via the intracellular NF-κB signaling machinery) and the release of TNF-α, supported by data from *in vivo* tumor models (Fischer et al., 2013; Cabrera-Fuentes et al., 2014; Han et al., 2019; Tielking et al., 2019). This reaction requires specific intracellular proteolytic processing as well as cleavage of the premature transmembrane form of TNF-α by TNF-α-converting enzyme (TACE, also denoted as ADAM17) (Figure 3). Here, exRNA was found to promote this shedding process to liberate the functionally active trimeric TNF-α from macrophages or other cells (Scheller et al., 2011). Since TACE recognizes and cleaves more than 50 cell membrane-anchored substrates other than pro-TNF (such as IL-6 receptor, VEGF-receptor 2, ICAM-1, E-selectin, NOTCH), the majority of which are directly or indirectly related to inflammation, it was proposed that exRNA could serve as a universal DAMP or alarmin, triggering such events at an early time point and at any site in the body (Zunke and Rose-John, 2017; Lambrecht et al., 2018). Yet, further experimental proof for such molecular mechanisms that would govern the exRNA-TNF-α axis or other TACE-related inflammatory reactions is still needed.

When exposed to external stimuli, monocytes/macrophages respond with rapid changes in the expression of various inflammation-related genes while undergoing polarization toward the M1-like (pro-inflammatory) or the M2-like (anti-inflammatory) phenotype. While both, extracellular microRNAs and long-non-coding RNAs could influence macrophage polarization, associated with “meta-inflammation” (Li et al., 2018), the exposure of murine bone marrow-derived macrophages (which were differentiated with mouse macrophage colony-stimulating factor) toward ribosomal exRNA resulted in their robust polarization toward the M1-like phenotype. A variety of typical M1 markers, including TNF-α, iNOS, IL-1β, or IL-6, were highly expressed, whereas anti-inflammatory genes, such as macrophage mannose receptor-2 (CD206) and other M2-markers, were significantly downregulated (Cabrera-Fuentes et al., 2015a). Likewise, treatment of human peripheral blood monocytes with exRNA followed by microarray analyses of the whole human genome revealed an appreciable upregulation of more than 70 genes, many of which are coupled to inflammation and the related signal transduction. Since the described macrophage responses to exRNA are independent of TLR3- or TLR7/TLR8-related signaling pathways (Cabrera-Fuentes et al., 2015a), it is fair to assume that ribosomal exRNA-mediated cytokine mobilization is largely independent of TLR-induced signaling, as was already noted for the transmigration of leukocytes (Zernecke and Preissner, 2016; Fischer, 2018). However, the typical TLR3-agonist poly (I:C) was capable of inducing M1-like polarization of tumor-associated macrophages, thereby inhibiting the tumor growth in a subcutaneous transplantation tumor model (Liu et al., 2016).

Together, these relationships are consistent with a still hypothetical but possibly general type of exRNA-dependent endogenous inflammatory cascade that begins with the liberation of exRNA (as a universal alarmin or DAMP) at a damage or infection site in any tissue of the body. Based on the ubiquitous expression of TACE, exRNA may induce the production of inflammatory products derived from proteolytic cleavage of the corresponding substrate(s) by TACE in a cell type-specific manner (as described for macrophage TNF-α). Thus, the “non-specific” alarmin exRNA could be capable of promoting “site-specific” cellular responses, some of which are of profound relevance for inflammation. Based on these considerations, not only is RNase1 an anti-inflammatory antagonist for exRNA-induced reactions, but TACE, the sheddase responsible for the release of TNF-α and other products (Preissner and Herwald, 2017), appears to be a considerable target as well. In fact, the TACE inhibitor TAPI has been demonstrated to inhibit exRNA-mediated shedding of TNF-α in peripheral blood mononuclear cells as well as in different preclinical *in vivo* models of cardiovascular disease, such as cardiac ischemia-reperfusion injury (Fischer et al., 2012; Cabrera-Fuentes et al., 2014). The increased adhesion of leukocytes to endothelial cells induced by exRNA *in vivo* was also attenuated by TAPI (Fischer et al., 2012).

Tissue-resident, perivascular mast cells are well known for their role as primary DAMP- or PAMP-sensing immune cells in inflammatory responses as well as for their immediate response in allergic and anaphylactic reactions. Their contribution to the process of arterial remodeling will be discussed below. In mature mast cells, secretory granules are located in close proximity to ribosomes and cytosolic ribosomal RNAs, and following cell activation and degranulation, these are released together with the content of granules (such as cytokines, lipid mediators, vasoactive substances) (Dvorak et al., 2003). *In vitro*, various agonists were found to induce the degranulation of mast cells and the concomitant release of appreciable amounts of EV-associated exRNA (Elsemuller et al., 2019). Although exRNA is not located in granules, the liberation of exRNA can be prevented by mast cell stabilizers or by abolishing the increase of intracellular Ca^2+^ levels in these cells. Mast cell-derived and EV-associated exRNA was further shown to promote the increased expression and release of cytokines (such as MCP-1 or IL-6) in vascular endothelial cells in a dose-dependent manner. These data indicate that exRNA-containing EVs from mast cells are likely to be involved in inflammatory responses and support earlier observations on the pivotal role of EVs in inflammation (Chen et al., 2019). However, which mechanistic route such EV-associated exRNA may take to activate and even enter target cells, possibly by using connexin-43 hemi-channels (Willebrords et al., 2016), needs to be further investigated.

### ExRNA and Arteriogenesis: A Blueprint for the Creation of Natural Vessel Bypasses Through Innate Immunity Reactions

**FIGURE 4:**
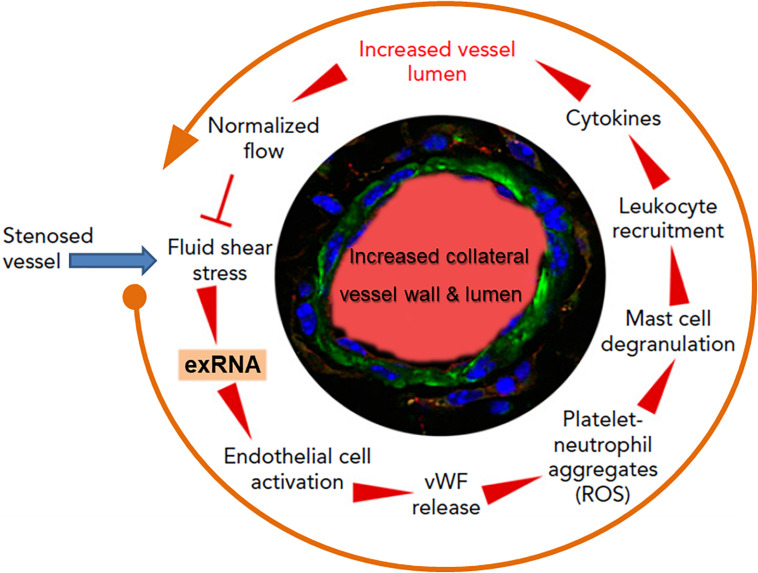
The multi-step cycle of exRNA-initiated arteriogenesis: a blueprint for blood vessel regeneration based on leukocyte recruitment. Upon occlusion of a feeding artery (e.g., in the heart), blood flow is redirected into collateral blood vessels that are formed by arteriogenesis from pre-existing arterioles within several days, thereby circumventing the stenosed artery. Due to the initially disturbed blood flow causing increased fluid shear stress, exRNA is released from endothelial cells and serves as a mechano-transducer, provoking further activation of vascular cells (in a VEGF-dependent manner). Thus, activated endothelial cells liberate von Willebrand factor (vWF) and other components from their Weibel–Palade granules, leading to platelet activation and formation of platelet-neutrophil aggregates with the production of reactive oxygen species (ROS). These, in turn, are essential for the activation of perivascular mast cells with the release of vasoactive factors (such as histamine) and chemo-attractive cytokines, a prerequisite for the recruitment of leukocytes that boost vascular cell proliferation by supplying growth factors and cytokines to promote collateral vessel growth. When collaterals reach a critical size of their lumen and vessel wall, allowing them to substitute for the function of the occluded artery, blood flow is normalized, and the collaterals cease to grow. Modified from Lasch et al. (2019).

Collateral artery growth, defined as arteriogenesis, is the only natural way for the body to spontaneously create blood vessel bypasses to counteract and circumvent the disastrous consequences of arterial occlusion, as in association with myocardial infarction (Faber et al., 2014). The initial trigger for this multistep intricate process is an increased fluid shear stress in pre-existing arterioles; this mediates endothelial cell activation, leukocyte recruitment, cytokine release, and subsequent endothelial and smooth muscle cell proliferation to finally induce controlled blood vessel expansion (Pipp et al., 2004; Lasch et al., 2019). Yet, how these different steps all work together for blood vessel regeneration remained unanswered for a long time (Deindl and Schaper, 2005). Based on the recent findings that exRNA is liberated from shear stress-exposed endothelium, we hypothesized that exRNA may serve as a trigger and promoter of collateral vessel growth, including endothelial activation, leukocyte recruitment and cytokine release as well as stimulation of the VEGF signaling axis (Kluever et al., 2019; Lasch et al., 2019; Figure 4).

As investigated in appropriate animal models, elevated fluid shear stress proximal to the occluded vessel(s) leads to the release of exRNA from endothelial cells and to platelet activation. Together, these reactions promote the exocytosis of Weibel–Palade granules with the release of von Willebrand factor from endothelial cells in a VEGF-receptor 2-dependent manner (Chandraratne et al., 2015; Lasch et al., 2019). In turn, von Willebrand factor initiates further activation of platelets and the formation of platelet-neutrophil aggregates, which release several pro-inflammatory agonists, including ROS, in the perivascular zone. Subsequent activation and degranulation of perivascular mast cells, which appear to orchestrate the extravascular reactions, result in the local increase and bioavailability of pro-inflammatory cytokines such as TNF-α and MCP-1, feeding a positive-feedback loop for the recruitment of more neutrophils, additional monocytes, and T cells (Chillo et al., 2016). Together, these factors all promote vascular cell proliferation and positive outward remodeling of the vessel wall, finally resulting in an arterial bypass that compensates the dysfunction of an occluded artery. The different steps of leukocyte recruitment appear to be a blueprint for arteriogenesis taken from the respective steps in innate immunity, whereby exRNA is considered in this context not to be a damaging component but rather a vessel and tissue regenerating factor. Although the aforementioned process of collateral artery growth also entails reactions of vasculogenesis and angiogenesis at distant sites, a definitive role of exRNA here is still speculative (Lasch et al., 2020). Nevertheless, in an *ex vivo* cellular spheroid model of vasculogenesis, exRNA was found to stimulate the formation of new vessels and leukocyte differentiation in embryonic bodies via increased VEGF-dependent signaling and ROS production (Sharifpanah et al., 2015). The underlying mechanisms remain to be described.

## exRNA and Vascular Defense Systems (Contact Phase/Coagulation, Blood Pressure Regulation)

Based on the recent characterization of new humoral and cellular factors, hemostasis as part of the wound healing process has gained considerable interest as contributor to the body’s life-saving defense mechanism that is embedded in the functional context of innate immunity. Here, self-exRNA and -exDNA were found to be potent cofactors in the initiation of blood coagulation, whereby the neutrophil-derived exDNA/histone scaffolds (designated as NETs) not only serve as potent anti-inflammatory gate-keepers that catch and kill microbes but also work as promoters of thrombotic situations in a variety of diseases (Fuchs et al., 2010; Massberg et al., 2010; von Brühl et al., 2012; Döring et al., 2017; Sorvillo et al., 2019; Thålin et al., 2019; Thiam et al., 2020). Since the identification of exRNA as a cofactor for several coagulation and platelet-derived proteins, the exRNA-mediated link between the discussed processes of innate immunity/leukocyte recruitment and the hemostasis system has become apparent.

The four plasma proteins factor XII, factor XI, prekallikrein, andHMWK are designated as “contact phase proteins” because they bind with high affinity to negatively charged surfaces on which they eventually become (auto-) activated and/or they reciprocally activate each other in a zinc ion-dependent manner (Kannemeier et al., 2007; Schmaier, 2016). Under *in vitro* settings or conditions of severe trauma *in vivo* this self-amplifying system promotes the intrinsic pathway of blood coagulation, culminating in thrombin generation and fibrin clot formation (Schmaier, 2016; Amiral and Seghatchian, 2019). Bioactive surfaces that promote contact phase activation include the activated endothelium and platelets, leukocytes, bacteria, and denatured proteins. Although factor XII appears to be dispensable for physiological hemostasis, under pathological situations associated with thrombotic complications the contact phase system contributes to enhanced clot formation, particularly in arterial thrombosis (Maas et al., 2011). This appears to be due to exposed collagens, sulfatides, platelet-derived polyphosphates, and misfolded proteins as well as exRNA and exDNA, which become accessible under conditions of severe trauma or inflammation or upon vascular pathologies and thereby serve as major natural activators or cofactors of contact phase activation (Kannemeier et al., 2007; Björkqvist et al., 2014; Naudin et al., 2017). These poly-anionic molecules induce auto-activation of factor XII and factor XI with major consequences for enhanced thrombin formation and thrombosis; they also promote selective generation of kallikrein, which in turn induces bradykinin formation from HMWK, relevant for vasodilation in the context of inflammation and edema (Schmaier, 2018). In order to prevent uncontrolled clotting, histidine-rich glycoprotein in plasma has been identified and characterized as exRNA- and factor XIIa-binding protein to attenuate their capacity to trigger coagulation (Vu et al., 2015). In fact, carotid artery occlusion was accelerated in histidine-rich glycoprotein-deficient mice which could be counteracted for by RNase administration as indicated above, supporting the pro-thrombotic role of exRNA in hemostasis. Independent of its function in coagulation, factor XII/XIIa exerts mitogenic activity in vascular cells, upregulates neutrophil functions, contributes to macrophage polarization, and induces T-cell differentiation (Schmaier and Stavrou, 2019). Thus, the exRNA-factor XII axis not only influences hemostasis and wound repair but may also contribute to other reactions in innate immunity (Bender et al., 2017; Renné and Stavrou, 2019).

In this context it is worthwhile mentioning that complex formation of exRNA (or exDNA) with basic platelet proteins such as platelet factor 4 (exposed during trauma, surgery, or infection) induces neo-epitopes in this basic protein that provoke the formation of autoantibodies, designated as HIT (“Heparin-induced thrombocytopenia”) antibodies (Jaax et al., 2013). Once the titer of these HIT antibodies increases, e.g., by administration of heparin in the same patient several years later, a thrombo-embolic scenario is generated due to the autoimmune character of HIT with the formation of micro-thrombi and the reduction of circulating platelets (Greinacher et al., 2017). These data indicate that exRNA (and other poly-anions) can induce autoimmunity in connection with the adaptive part of the immune system. Moreover, additional pathological situations have been recognized in neurological disorders in association with cellular damage, where autoantibodies against intracellular RNA-binding proteins have been recognized in plasma, possibly contributing to the onset or progress of autoimmune diseases such as Opsoclonus-Myoclonus syndrome (Blaes et al., 2007).

## The Extracellular RNA/RNase System

The lifespan as well as the reactivity of exRNA species in the vasculature or in other extracellular body fluids depends to a large degree on the type of complexes formed with proteins, EVs, or cell surfaces. Despite these constraints, exRNA is continuously degraded in the vascular system by circulating RNases, of which the thermo-stable RNase1 is the by far most powerful natural antagonist of exRNA. RNase1 belongs to the RNaseA family, which consists of eight members that have endonuclease activities and which are secreted by a large variety of different tissues and cells (Koczera et al., 2016). Eosinophil (RNase 2, RNase3) or epithelial cell-derived RNase7 serves as an anti-microbial protein, whereas RNase5 (also designated angiogenin) has potent angiogenic functions without having an appreciable ribonucleolytic activity (Cho et al., 2005; Sorrentino, 2010). Extracellular RNases can also be internalized by cells via endosomal pathways (Haigis and Raines, 2003), but due to the action of RNase inhibitor, which binds mammalian RNaseA family members with an extremely high affinity, these endonucleases are immediately inactivated and do not express any intracellular cytotoxic activity (Dickson et al., 2005; Rutkoski and Raines, 2008).

Pancreatic RNase1 is produced in the exocrine pancreas and constitutes the major ribonuclease of the gastrointestinal tract, whereas vascular RNase1 is predominantly expressed and released by endothelial cells from medium and large vessels as well as in the umbilical vein (Landré et al., 2002; Barrabés et al., 2007; Fischer et al., 2011; Eller et al., 2014; Ohashi et al., 2017). Interestingly, the counteracting function of RNase1 that, as we reported in various preclinical cardiovascular models, combats the damaging functions of exRNA is robust and safe due to the fact that RNase inhibitor with its extremely high affinity for RNases is present in all cell types of the body (Arnold, 2011). Thus, RNase1 bears considerable potential as new therapeutic agent based on its tissue- and vessel-protective functions that all may translate into anti-inflammatory properties in different pathological situations (Cabrera-Fuentes et al., 2015b; Kleinert et al., 2016; Zernecke and Preissner, 2016; Ma et al., 2017; Stieger et al., 2017; Table 1).

**TABLE 1:** ExRNA-counteracting properties of RNase1 as studied *in vitro* and in preclinical disease models.

Biological system, pathological process	Influence/activity of RNase1	References
Blood coagulation, thrombosis	Destruction of exRNA as cofactor for coagulation proteases; anti-thrombotic	Kannemeier et al., 2007
Vascular hyperpermeability, vasogenic edema formation, stroke	Destruction of exRNA as cofactor for VEGF; vessel-protective	Fischer et al., 2007; Walberer et al., 2009
Inflammation	Destruction of exRNA as cytokine cofactor; anti-inflammatory	Bedenbender and Schmeck, 2020
Tumor growth in xenograft, immuno-compromised model	Destruction of exRNA as triggering factor for promoting tumor cell trafficking; anti-tumorigenic	Fischer et al., 2013
Atherosclerosis, arterial vessel degeneration	Destruction of exRNA as multifunctional cell-damaging factor; anti-atherogenic	Simsekyilmaz et al., 2014
Cardiac ischemia-reperfusion injury, heart failure	Destruction of exRNA as cardiomyocyte-damaging and cytokine-mobilizing factor; anti-cytotoxic, cardio-protective	Cabrera-Fuentes et al., 2014
Experimental heart transplantation	Prolongation of graft survival; tissue-protective	Kleinert et al., 2016
Microbial infection	Prevention of exRNA-mediated bacterial adherence and invasion; anti-microbial	Zakrzewicz et al., 2016
Myocardial infarction	Reduction of cardiac edema and infarct size; cardio-protective	Stieger et al., 2017
Shear stress-mediated induction of arteriogenesis	Reduction of collateral vessel formation; anti-inflammatory	Lasch et al., 2019

Different stimulatory agonists or conditions such as pro-inflammatory or pro-thrombotic agents, exRNA itself, vasopressin as well as ischemic conditioning may induce the short-term release of RNase1 from its endothelial storage sites, the Weibel–Palade granules. Consequently, the exRNA/RNase1 system can be considered as an integral regulatory part of vascular homeostasis, vessel wall integrity, and the innate immune response, including the recruitment of leukocytes (Fischer et al., 2011; Zernecke and Preissner, 2016; Lomax et al., 2017). Under inflammatory conditions, however, such as long-term stimulation by thrombin or TNF-α, the expression and protein synthesis of the protective RNase1 in endothelial cells was found to be repressed as a result of epigenetic mechanisms (Gansler et al., 2014; Bedenbender et al., 2019). The inhibitory and protective effects of RNase1 are most likely due to the degradation and the elimination of damaging exRNA species; whether the hydrolysis products of exRNA, such as (oligo-) nucleotides or nucleosides (as vaso- or neuro-active compounds) that are generated, may contribute to the overall protective function of RNase1 in the body remains to be investigated. Studies of plasma from RNase1-deficient mice demonstrated that its pro-coagulant status was much higher than the plasma of wild-type mice, which confirmed that one of the physiological functions of RNase1 is to degrade exRNA in blood plasma and to serve potent anticoagulant functions (Kannemeier et al., 2007; Garnett et al., 2019). RNase1 is furthermore involved in immune responses by inducing phenotypic and functional maturation of dendritic cells and viral defense mechanisms, e.g., in the inactivation of HIV (Lee-Huang et al., 1999; Yang et al., 2004).

Based on the different exRNA/inflammation-driven preclinical pathological situations discussed above, in a rat model of cerebral stroke initiated by *Sinus sagittalis* thrombosis, the edema formation and infarct size were significantly reduced after pretreating the animals with RNase1 but not with DNase, confirming the permeability-increasing activity of exRNA *in vivo* (Fischer et al., 2007). Likewise, in a preclinical mouse model of myocardial infarction (ligation of the left anterior descending coronary artery), increased levels of exRNA were found to provoke myocardial edema formation 24 h after ligation as compared with controls. Consequently, the systemic application of RNase1 (but not DNase) markedly increased the area of vital myocardium (Stieger et al., 2017). Thus, RNase1 efficiently counteracts exRNA-induced edema formation and preserves perfusion of the infarction border zone, resulting in a reduction of infarct size and the protection of cardiac function after myocardial infarction. Finally, successful translational approaches are underway to target the adverse functions of extracellular nucleic acids in patients by using nucleic-acid binding polymers such as poly-amidoamine dendrimers as novel anti-inflammatory and anti-thrombotic drugs (Lee et al., 2011; Holl et al., 2013; Naqvi et al., 2018). Together, these approaches underline the utmost importance of further defining and understanding the (patho-) physiological role of exRNA in immune defense.

## Perspectives and Hypotheses: Possible Contributions of exRNA in Spreading Cardiopulmonary and Vascular Diseases in Association With COVID-19

**FIGURE 5:**
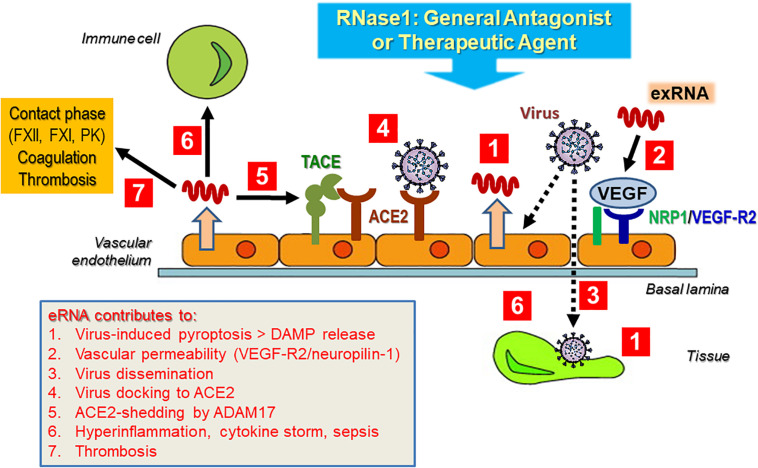
Hypothetical contribution of exRNA to virus infection and subsequent cardiovascular disease. Upon infection of the tissue (vascular endothelium) by (SARS-CoV-2) virus, DAMPS including exRNA are released from tissue cells (Tisoncik et al., 2012; Castanheira and Kubes, 2019). Virus particles activate tissue and immune cells of the host to release inflammatory mediators, including exRNA, which in turn can induce vascular hyper-permeability in a VEGF-receptor-2-neuropilin-1 (NRP1)-dependent manner (Van Kaer et al., 2013). This allows virus particles to further invade particular tissue sites to disseminate their infection potential, including the liberation of cytokines (Kolaczkowska and Kubes, 2013; Zindel and Kubes, 2020). A major docking site for SARS-Cov-2 is the endothelial cell-associated metalloproteinase ACE2, whose processing, carried out by ADAM17, could be triggered by exRNA to allow virus entry (Bianchi, 2007; Andersson and Tracey, 2011). Together, these mostly hypothetical exRNA-mediated processes not only could provoke a robust cytokine release, associated with hyper-inflammation and cardiovascular disease. In addition, exRNA as a pro-thrombotic cofactor can further induce the intrinsic pathway of blood coagulation, resulting in the thrombotic phenotype of COVID-19 patients. As a general antagonist or potential therapeutic agent, RNase1 may inhibit or prevent the multiple actions of the damaging exRNA, associated with viral infections and their adverse side effects. For details please see text.

At present, a detailed characterization of the interactions of exRNA with viruses in general and with severe acute respiratory syndrome-related coronavirus 2 (SARS-CoV-2) in particular is pending; however, as alluded to in the topics discussed here, self-exRNA may serve a prominent role in promoting and disseminating a viral infection and its subsequent adverse side effects in the human host (Figure 5). This hypothesis is based on the fact that virus infections in particular are known to damage and destroy host cells with the liberation of a number of DAMPs and that cardiovascular diseases appear to be serious adverse effects of coronavirus infections. In fact, HMGB1 or NETs could serve as potential targets for therapies in coronavirus disease (COVID-19) (Andersson et al., 2020; Cicco et al., 2020).

Severe acute respiratory syndrome-related coronavirus 2 is a positive-sense, single-stranded enveloped RNA virus responsible for the ongoing COVID-19 that initially appeared in China in 2019 (Perlman and Netland, 2009; Huang et al., 2020; Ren et al., 2020; Zhu et al., 2020). Several types of coronaviruses with a zoonotic potential have been described to infect humans and animals. While those viruses mainly infecting the upper respiratory tract cause only minor symptoms, the coronaviruses infecting the lower respiratory tract (with SARS-CoV-2 and MERS-CoV among them) can also provoke fatal pathological side effects associated with cardiovascular diseases, with an estimated mortality rate between 3 (SARS-CoV-2) and 35% (MERS) (Labò et al., 2020; Tay et al., 2020). The case fatality for COVID-19 caused by SARS-CoV2 has been variably estimated between <1 and 15% (Rajgor et al., 2020). ARDS, sepsis, and multi-organ failure involving the kidney and heart were described as causes of mortality in the majority of severe SARS-CoV-2 infections (Chen et al., 2020; Goh et al., 2020; Wu and McGoogan, 2020). Accordingly, it is of major interest to understand the fundamental molecular and immunological mechanisms of SARS-CoV-2-caused pathologies, and identifying effective drugs for treating patients and combating viral infection will be critical.

To initiate a viral infection with SARS-CoV-2, the protein spike subunit S12 is recognized by several host cell-surface receptors, the prominent one being angiotensin-converting enzyme 2 (ACE2), which triggers the endocytosis of the virus (Tay et al., 2020). To facilitate host cell entry, the virus engages the cellular transmembrane protease serine 2 (TMPRSS2), which processes the viral spike protein, a prerequisite for coronavirus entry (Hoffmann et al., 2020). TMPRSS2 is also able to cleave ACE2 and thereby competes with the sheddase TACE: While TACE-induced proteolysis is associated with the release of TNF-α, only TMPRSS2-mediated shedding appears to augment the amplification of viral entry upon SARS-CoV-2 infection (Haga et al., 2008; Heurich et al., 2014). Here, it would be relevant to analyze the influence of exRNA on these proteolytic processing events, since we have proposed that there is a direct effect of exRNA on triggering TACE to augment substrate cleavage (Fischer et al., 2013). Moreover, exRNA was shown to directly affect the (auto-)activation of proteases in blood plasma such as contact phase enzymogens or factor VII-activating protease (Nakazawa et al., 2005; Fischer et al., 2013).

Recent data indicated that neuropilin-1 significantly potentiates SARS-Cov-2 infectivity, implying a particular role in viral pathogenicity for this co-receptor of VEGF-receptor-2 (Cantuti-Castelvetri et al., 2020; Moutal et al., 2020). Based on the expression of neuropilin-1 in endothelial and epithelial cells of the olfactory and respiratory system, its upregulation in SARS-CoV-2-infected blood vessels of COVID-19 patients was associated with vascular endothelialitis, angiogenesis, and thrombosis (Ackermann et al., 2020). Considering the previously described relations of exRNA to these components in provoking vascular permeability, angiogenesis, and inflammatory reactions, it is fair to propose an influence of exRNA on the VEGF-receptor-2/neuropilin-1 system in the context of virus infection, thereby enhancing the leakiness of blood vessels and promoting dissemination of virus and its penetration into tissues (Figure 5).

Cellular infections by cytopathic viruses such as SARS-Cov-2 cause cell damage and pyroptosis, a highly inflammatory form of cell death, which results in release of DAMPs such as cellular nucleic acids, including self-exRNA (Tay et al., 2020). These inflammatory agonists are recognized by neighboring tissue and immune cells to trigger the release of pro-inflammatory cytokines and chemokines, including IL-6, IFN-γ, and MCP-1 as described (Tay et al., 2020). In this context, exRNA as well could amplify this response, also by attracting leukocytes and other immune cells in a VEGF-receptor-2-dependent manner, as demonstrated in several preclinical studies (Fischer et al., 2012; Kluever et al., 2019; Lasch et al., 2019). In the ultimate phase of inflammation under physiological conditions, the attracted neutrophils and macrophages need to clear the site of infection/inflammation by phagocytosis and induce the final inflammatory step of resolution and recovery. In patients with severe COVID-19, however, a dysfunctional immune response results in further mobilization of immune cells causing an amplification of the cytokine storm with fatal consequences not only for the lung but also for other organs (Huang et al., 2020; Sariol and Perlman, 2020). Based on the observed cell damage under these pathological conditions, it might be speculated whether exRNA is involved in this self-amplifying cytokine storm and whether administration of RNase1 might help to suppress this fatal scenario (Figure 5).

There might be another, yet undefined connection between exRNA and the systemic cytokine storm, hemorrhage, and sepsis that are the major causes of death in COVID-19 patients. This potential connection is based on the role of “cold-inducible-RNA binding protein” (CIRP) and its relevance for community-acquired pneumonia, whose severity is related to microbial pathogenicity and virus load (Guo et al., 2020). As a typical DAMP, nuclear CIRP is liberated by macrophages and other cells upon stress and promotes inflammatory responses (cytokine release) via the TLR4-myeloid differentiation factor 2 complex, also causing endothelial dysfunction (Qiang et al., 2013; Figure 1). The blockade of CIRP using antisera to CIRP has been shown to attenuate inflammatory cytokine release and mortality after hemorrhage and sepsis. CIRP was also documented to induce NET formation, which causes tissue damage in lungs during sepsis, whereas for COVID-19 patients it was noted that organ dysfunction was due to vascular occlusion by NETs (Ode et al., 2019; Leppkes et al., 2020). Interestingly, injection of CIRP into mice caused vascular leakage, edema formation, and leukocyte infiltration with cytokine production in the lungs that was accompanied by endothelial cell activation and pyroptosis (Yang et al., 2016). Altogether, these observations are reminiscent of the multiple functions of exRNA as a DAMP and damaging factor, as summarized in this review, suggesting that CIRP and exRNA might work together and amplify each other as potent inflammatory companions, as was demonstrated for TLR2-ligands and exRNA (Noll et al., 2017). In fact, complexes of DAMPs such as HMGB1 or CIRP with exRNA could drastically enhance the stimulatory function of each factor, e.g., to induce TNF-α expression and release in macrophages (Andersson et al., 2020). Moreover, treatment of mice suffering from septic cardiomyopathy with RNase1 resulted in a reduction of cardiac apoptosis, TNF-α expression, cardiac injury, and dysfunction (Zechendorf et al., 2020). Together, these data demonstrate that exRNA could play a crucial role in the patho-physiology of organ dysfunction in sepsis, a very critical situation in COVID-19 patients.

The observed vascular damage in SARS-CoV-2 infections is related to virus-mediated endothelial cell injury that exacerbates endothelial dysfunction, as it is known from aging, hypertension, and obesity, and which is likely to be associated with the complications and mortality observed in COVID-19 patients (Amraei and Rahimi, 2020). Endothelial dysfunction in COVID-19 is linked to hypercoagulability as indicated by increased fibrinogen and von Willebrand factor levels as well as elevated numbers of activated platelets and their complexes with leukocytes together with abnormal coagulation parameters (Spiezia et al., 2020; Tang et al., 2020b). Whether exRNA and other procoagulant DAMPs such as NETs might directly or indirectly worsen this thrombogenic situation in COVID-19 patients remains to be confirmed (Thierry and Roch, 2020). In order to tackle a given thrombotic risk situation, anticoagulants such as heparin have been successfully administered as versatile drugs to treat COVID-19 patients to reduce their mortality rate (Tang et al., 2020a; Thachil, 2020). The high affinity of different types of heparin for the SARS-CoV-2 spike protein appears to be relevant for these poly-anions to interfere with the mechanism of virus entry into host cells (Kwon et al., 2020). Although heparin and exRNA are known to compete in binding to several cytokines and growth factors that contain basic heparin binding sites, data showing competition between exRNA and virus proteins are not available (Fischer et al., 2007). Taking these findings together, one can speculate that elimination of exRNA as an endogenous prothrombotic cofactor by RNase1 might help to reduce the adverse thrombotic complications in COVID-19 patients.

Finally, the involvement of vascular components like ACE2 as the SARS-CoV-2 entry site of host cells has a profound influence on the homeostasis of blood pressure regulation via the renin-angiotensin-aldosterone system. Not only does virus docking to ACE2 cause its masking and downregulation, but there is also a concurrent loss of protease activity to generate angiotensin (Bianchi, 2007; Andersson and Tracey, 2011; Tisoncik et al., 2012; Kolaczkowska and Kubes, 2013; Van Kaer et al., 2013; Castanheira and Kubes, 2019; Zindel and Kubes, 2020) from angiotensin II (Amraei and Rahimi, 2020). This leads to a substantial drop in the intrinsic control of the hypertensive arm of the system, resulting in cardiovascular complications in COVID-19 patients. Moreover, ACE2 is expressed in endothelial cells of capillaries, arterioles, arteries, and veins in many organs of the body, and ACE2 knockdown in mice was found to be associated with increased expression of inflammatory cytokines, metalloproteinases, and endothelial adhesion receptors, all molecules that are relevant for leukocyte recruitment (Hamming et al., 2004; Thomas et al., 2010). This would indicate that a reduction or loss of ACE2 under conditions of SARS-CoV-2 infection might lead to an accentuation of vascular inflammation and even atherosclerosis. However, in retrospective studies no higher risk of infection in patients with ACE inhibitors was found (Chung et al., 2020). Whether these critical situations can be counteracted in COVID-19 patients by intervention with RNase1 as described above remains to be investigated. Nevertheless, at different steps and the respective host responses during a SARS-CoV-2 infection, there are several scenarios where exRNA appears to be involved or serves as a virulence factor, and the antagonistic role of RNase1 may provide general anti-viral and tissue-protective functions. Further research activities are needed to prove this hypothesis.

## Author Contributions

All authors contributed to the design and the production of the review article.

## Conflict of Interest

The authors declare that the research was conducted in the absence of any commercial or financial relationships that could be construed as a potential conflict of interest.

## Footnotes

1 http://exRNA.org/
